# 22q11 Deletion Syndrome and Multiple Complex Developmental Disorder: A Case Report

**DOI:** 10.4021/jocmr1222w

**Published:** 2013-02-25

**Authors:** Valeria Scandurra, Maria R. Scordo, Roberto Canitano, Esther I. de Bruin

**Affiliations:** a Department of Child Neuropsychiatry, University of Florence; Viale Pieraccini - 50139, Firenze, Italy; b Department of Child Neuropsychiatry University Hospital of Siena; Viale Bracci 1-53100, Siena, Italy; c University of Amsterdam, Faculty of Behavioral and Social Sciences, Research Institute of Child Development and Education, Nieuwe Prinsengracht 130, 1018 VZ, Amsterdam, the Netherlands

**Keywords:** Autism, Pervasive Developmental Disorder-Not Otherwise Specified Line, Genetics, 22q11 deletion syndrome, Multiple Complex Developmental Disorder

## Abstract

22q11.2 Deletion Syndrome (22q11 DS) is a multisystemic condition that may also include neuropsychiatric disorders. We present a case of a 15-year-old boy that was evaluated for social difficulties, and anxiety with the above genetic abnormality. Clinical features were rather complex as different neuropsychiatric symptoms emerged from assessment and clinical evaluation. As a result we propose that Multiple Complex Developmental Disorder (MCDD) would be the best fitting comprehensive diagnosis to be added to the DSM-IV category of Pervasive Developmental Disorder-Not Otherwise Specified.

## Introduction

The 22q11.2 deletion syndrome (22q11DS) is a multisystem disorder caused by a microdeletion from chromosome 22 at the q11.2 band. It is also known as velo-cardio-facial syndrome and its prevalence is estimated 1:2000 - 7000 live births [1]. The majority of micro deletions occurring de novo in the 90% and remaining 10% is inherited [2]. This syndrome has a variable phenotype with many clinical features described that could involve many organs, systems and developmental functions. Cognitive, behavioral and psychiatric symptoms are very common: Attention-Deficit-Hyperactivity Disorder (ADHD) and Mental Retardation (MR) have been described [3]. Autism Spectrum Disorders (ASD) were studied and estimated to be prevalent in 14-33% of the cases [4-6]. The ASD phenotype in 22q11DS was found more heterogeneous than in idiopathic ASD [7]. Furthermore, schizophrenia, psychotic symptoms and disorders are described in a relevant number of affected patients ranging from 20% to 33% [6, 8]. Vorstman et al [6] evaluated 60 patients with 22q11DS and found 33% to meet criteria for ASD as measured with the Autism Diagnostic Interview-Revised (ADI-R) [9] and 26.7% to suffer from psychotic symptoms. The authors conclude that ASD and psychotic disorders should be considered relevant clinical features of 22q11DS subjects.

## Case Report

### Clinical history

The patient is a Caucasian boy born from healthy unrelated parents. He also has a healthy younger sister. The pregnancy was uneventful but he was born by emergency caesarean section at 39 weeks because of reduced fetal heartbeat. Meconium liquor was present. At birth rapid recovery was noted with Apgar score 8-10; weight 2.2 kg (< 5th percentile), and at neurological evaluation, mild hypotonia was observed. Growth rate was between the 10 - 50th percentiles during the first 24 months and later it stabilized on the 50th percentile. Bowel control was obtained at 48 months. Nocturnal enuresis was present until seven years. Psychomotor development milestones were slightly delayed with sitting at about 11 months and walking at the age of 18 months. He presented mild general hypotonia, hypoactivity and fatigue throughout the day. At twelve years he developed a migraine with aura.

With respect to language and communication development he started to pronounce his first words at 12 months and complete phrases at 24 months; pointing at objects was present at 30 months, while the other communicative gestures including declarative, emotional and conventional gestures were poor. He developed literal comprehension of language with difficulties to understand metaphorical sentences.

At age 15 (his current age) interaction with peers is characterized by scarce initiative, without trying to get their attention. Poor social awareness and interaction with peers became more evident during late childhood and adolescence. Prominent difficulties were observed in establishing and maintaining a direct and empathetic relationship with peers over time. Currently, he has difficulties in modulating social interaction, for example, he shows excessive intrusiveness, or he avoids peers. However, in interaction with adults, his behavior is more appropriate.

With respect to playing and using objects, he developed a selective interest in a limited range of things, for example, he has a special interest for collecting CD’s of a single artist. Also, he has a specific interest for geography. Further, he developed an excessive anxiety concerning separation from his parents, at the same time he sometimes shows aggressive behavior towards his family members.

### Clinical evaluation

A 15-year-old boy with 22q11DS was evaluated in our clinic. At general evaluation he was in good health and did not complain of any symptoms. At physical examination, hyperlaxity ligaments, flatfoot, were observed. He presented mild facial dimorphisms with long face, ocular hypertelorism, small ears, and a prominent nose with a bulbous tip. Muscular tone was found mildly reduced, fatigue was reported, and normal muscle trophism was found. The patient was able to walk and run. Global clumsiness and poor fine motor skills were observed. Genetic testing conducted with array-Comparative Genomic Hybridization (CGH) highlighted a pathogenic microdeletion of 1.5 Mb on the long arm of chromosome 22. Quantitative analysis using Multiple Ligation-Dependent Probe Amplification (MLPA) showed the following results: 46XY.mlpa 22q11.2.

His immunological profile demonstrated profile that demonstrated mild IgG- IgM reduction, mild lynphopenia, and hypo-γ-globulinemia was confirmed by electrophoresis. Other ematochemical investigation was found normal. General internist evaluation did not show additional pathological findings. Electroencephalogram (EEG) was normal. Brain Magnetic Resonance Imaging (MRI) was within normal limits.

### Neuropsychiatric assessment

Neuropsychiatric evaluation was completed by a senior neuropsychiatry clinician, a psychologist and a resident certified to administer the Autism Diagnostic Observation Schedule (ADOS) [10] and ADI-R.

#### Intellectual ability

Estimation of his general intellectual ability was obtained by administering the Wechsler Intelligent Scale for Children-version III [11]. Table 1 presents the details of his IQ scores. His Verbal Intelligence Quotient (VIQ) was significantly higher than his Performance Intelligence Quotient (PIQ). Factor scores were in line, that is, his scores on Verbal Comprehension (VC) subtests were significantly higher than those on Perceptual organization (PO) subtests. The cognitive profile of this patient was characteristic of the 22q11.2DS, both for IQ and factor scores [3, 12, 13].

**Table 1:** Neuropsychiatric Assessment

Test	Results
WISC-III	Full Scale IQ: 77; Verbal IQ: 90; Performance IQ: 69; Verbal Comprehension (VC): 93;
	Perceptual Organization (PO): 72;
	Freedom from distractibility (FD): 78;
	Processing Speed (PS): 85;
	QIV/QIP significant statistical difference 1%;
	CV > OP significant statistical difference 1%;
	CV > FD significant statistical difference 5%.
VABS-II	Adaptive Behavior Composite: 61;
	Communication: 59; Daily living skills: 63;
	Socialization: 64; Motor skills: 75;
	Maladaptive Behavior Scale index: clinically significant.
CBCL/4-18	Whitdrawn:88-C(> 98th);
Syndrome Scale Scores for boys 12 - 18	Somatic Complaints: 91-C(> 98th);
	Anxious/Depressed:81-C(> 98th);
	Social Problems: 87-C (> 98th);
	Thought Problems: 82-C(> 98th);
	Attention Problems:89C(> 98th);
	Aggressive Behavior: 68-B (97 th).
CPRS-R:L	Oppositional: 78; Cognitive/Inattentive: 69; Hyperactivity: 76; Anxious/shy: 97; Perfectionism: 62;
(T-Scores)	Social Problems: 100; Psychosomatic: 99;
	Conners’ ADHD Index: 79; I: CGI Restlessness-Impulsive: 71; J-CGI- Emotional Lability: 83; K-CGI Total: 76; L: DSM-IV Inattentive: 76; M- DSM-IV Hyperactive-Impulsive: 76; DSM-IV Tot: 79.
ADOS-Module 4	Communication: 4 (> cut-off autism);
	Social Interaction: 5 (> ASD);
	Total (Communication + Social Interaction): 9 (> ASD); Imagination: 1;
	Stereotyped Behaviors and restricted interests: 2.
ADI-R	Reciprocal Social Interaction: 14 (> ASD);
	Communication: 13(> ASD);
	Restricted, Repetitive, and Stereotyped Patterns of Behaviors:10 (> ASD);
	Abnormality of Development Evident at or before 36 Months: 3 (> ASD).
K-SADS-PL	Anxiety disorders: Separation Anxiety Disorder (SAD)

#### Adaptive functioning

An indication of his adaptive level was obtained by administering the Vineland Adaptive Behavior Scales- Second Edition [14]. Overall, his level of adaptive functioning was low for his age. His Maladaptive Behavior Scale index was clinically significant, see Table 1 for detailed VABS scores.

#### Externalizing and internalizing symptoms

His mother was asked to complete the Child Behavior Checklist 4-18 (CBCL) [15]; and the Conners’ Parent Rating Scales-Revised Long Version (CPRS-R:L) [16]. At the CBCL, several syndrome scales scored in the clinical and borderline range (Table 1). Scores on the broadband scales of Internalizing, Externalizing, and Total Problems are in the clinical range (> 98th, > 97th, > 98th percentile respectively). In line, on the CPRS-R:L an ADHD profile emerged with combined features of hyperactivity, impulsivity and inattention.

#### Communication and reciprocal interaction

For assessment of ASD symptoms the ADOS and ADI-R were administered. During ADOS assessment the patient showed inconstant eye contact, poor integration of verbal and non-verbal skills and limited empathy. Nonetheless he was able to start and participate in a social conversation, with fair narrative skills. However, he predominantly reported his paranoid preoccupations and unusual interests such as geography, and a specific kind of music. Also he showed unusually ritualized activities such as providing lists. The diagnostic algorithm of the ADOS scored positive for ASD (Table 1). The ADI-R was administered to the mother of the boy. As a preschooler, his mother described mild articulation difficulties, however she reported no language delay. His non-verbal skills were characterized by protodeclarative pointing and a limited range of gestures. Furthermore, he showed inconstant reactions to human voices. Many of the features in the past and the present were rated as ritualistic and obsessive behaviors.

#### Affective and psychotic symptoms

Clinical elements of Specific Phobia, Panic Disorder and depressive traits were reported. Moreover psychotic symptoms, magical thinking and persecutory religious thoughts were reported along clinical interview. For assessment of affective, psychotic and anxiety disorders, the Kiddie-Schedule for Affective Disorders and Schizophrenia Present and Lifetime version (K-SADS PL) [17] was administered. DSM-IV diagnostic criteria were met only for Separation Anxiety Disorder (Table 1).

## Discussion

The complex clinical features of the boy with 22q11DS suggested a comprehensive neuropsychiatric evaluation to highlight the different clinical components. To our knowledge, this is the first case where a complete ASD evaluation, including ADOS, has been administered in 22q11DS. This approach is important to define the specific social and communication characteristics in 22q11DS patients. Along the various steps of the evaluation many features emerged to support an ASD classification. Communication, and verbal language were poor, as well scarce non-verbal abilities interfered with social interaction. In addition, aspects of the third domain of ASD such as preoccupations and ritualistic behaviors emerged and were observed since early childhood. It is important to mention that limited social and communication skills are reported in the seminal paper on this topic by Vorstman et al [6] further emphasizing the ASD components in the 22q11DS. According to all the above clinical features, in DSM-IV [18] terms this boy would most likely meet criteria of Pervasive Developmental Disorder-Not Otherwise Specified (PDD-NOS). However, in this patient several psychiatric symptoms other than social and communication problems were found. At Conners’ profile he showed symptoms of ADHD such as inattention, hyperactivity, and impulsivity. Therefore overall clinical evaluation highlighted only moderate difficulties of attention principally interfering with learning and school performances. Furthermore several indications of anxiety, and psychosomatic symptoms including nausea, abdominal pain and fatigue were reported. Moreover thought problems were reported by the boy and by both his parents. Thus, the main three lines and complexity of clinical features of this boy consisted of impaired social behavior, emotional difficulties and psychotic thought problems. This is consistent with a clinical picture of Multiple Complex Developmental Disorder (MCDD) [19-21]. Children or adolescents with MCDD may suffer from peculiar fears, bizarre anxiety reactions, panic attacks, violent behaviors, magical thinking, bizarre ideas, paranoid preoccupations, as well as impaired social and attachment behavior. Since psychotic and paranoid thoughts are so characteristic of MCDD, these patients may have an increased risk for developing a psychotic disorder or even schizophrenia later in life [21]. MCDD is currently not a separate diagnostic entity in DSM-IV, but because of its pervasive nature, early onset, and deficits in multiple areas of development, the diagnostic classification mostly used for these patients is PDD-NOS [22]. However, even if this is the most elegant solution it covers a large debate in itself. For instance, it has been shown that not even half of the patients with MCDD meet full criteria for PDD-NOS [20]. A discussion about the positioning of MCDD as a construct falls beyond the scope of this case report. However our opinion is that the boy described in this case report would most comprehensively be classified under MCDD and further treatment would be tailored mainly to his psychotic thought problems and his difficulties with anxiety and affect regulation. If only ASD (possibly combined with ADHD or anxiety disorders) would be classified, following DSM-IV guidelines, it was felt that this patient would not be described in a fully accurate way and therefore treatment might not be adjusted to his more complex clinical picture. In previous reports of 22q11DS different psychiatric disorders have been described but this additional definition calls for carefully monitoring developmental trajectories. Whereas in previous studies the association between 22q11DS and symptoms of ASD and schizophrenia has been demonstrated, this case report illustrates a potential useful classification, MCDD, for these 22q11DS children in which both psychotic and autistic symptoms are combined. 22q11DS is one of the highest known risk factors for the development of schizophrenia [8] which might be in line with our observations, since children with MCDD have an increased risk of later development into a psychotic disorder or schizophrenia [21]. Further investigations are needed to clarify the complex phenotype of 22q11DS including the psychiatric characteristics.
